# Exercise activity, body size and premenopausal breast cancer survival

**DOI:** 10.1038/sj.bjc.6601820

**Published:** 2004-05-04

**Authors:** S M Enger, L Bernstein

**Affiliations:** 1 Department of Research and Evaluation, Kaiser Permanente Medical Care Program, Pasadena, CA 91101, USA; 2 Department of Preventive Medicine, University of Southern California/Norris Comprehensive Cancer Center, University of Southern California School of Medicine, Los Angeles, CA 90033, USA

**Keywords:** physical activity, BMI, weight, breast neoplasms

## Abstract

We evaluated prediagnosis predictors of breast cancer survival among 717 premenopausal breast cancer patients enrolled in a population-based case–control study and followed for 10.4 years. Using Cox's proportional hazards models, lifetime exercise, weight, and height were not associated with survival. Higher body mass index (trend *P*=0.21) and recent exercise activity (trend *P*=0.31) were weakly associated with longer survival.

Evidence suggests that physical activity reduces the risk of breast cancer (IARC, 2002), an effect first reported nearly 20 years ago (Frisch *et al*, 1985). In addition, high body mass and weight gain increase the risk of postmenopausal breast cancer (International Agency for Research on Cancer, 2002), though a weak inverse relationship between body mass and breast cancer risk has been noted among premenopausal women (Ziegler, 1997; Friedenreich, 2001; Morimoto *et al*, 2002).

As there is currently interest in whether breast cancer risk factors also impact on survival, we have examined these factors in a population-based cohort of breast cancer patients.

## MATERIALS AND METHODS

Subjects were breast cancer patients who participated in a population-based case–control study among women aged 40 years or younger (Bernstein *et al*, 1994). Briefly, eligible patients were white or Hispanic women consecutively diagnosed with their first primary *in situ* or invasive breast cancer from 1 July 1983 through 31 December 1989, identified by the University of Southern California Cancer Surveillance Program (CSP), the population-based cancer registry for Los Angeles County.

The University of Southern California institutional Review Board (Los Angeles, CA, USA) reviewed and approved the study that complied with all applicable federal regulations governing the protection of human subjects. Study subjects were given a detailed explanation of the study including the risk and benefits. All participants provided signed informed consent to participate in the study.

In face-to-face interviews, we obtained detailed information about demographics and known or suspected breast cancer risk factors, up to each patient's reference date, defined as 12 months before diagnosis.

We asked patients whether they had ever participated at least twice weekly on a competitive athletic team or in dance or exercise classes, or if they jogged or ran one mile at least twice weekly. For those who responded ‘yes’, we recorded for each activity the age started and stopped, the type, and the average number of hours per week of participation. We recorded each episode when activities were started and stopped more than once or when the amount of time spent in the activity changed. We computed the number of hours per week each patient participated in all recreational exercise activities beginning with the year of each woman's first menstrual period and ending at the reference date.

Details were collected of height and weight at the reference date and weight at age 18 years. Body mass index (BMI) was computed as weight (kg) divided by the square of height (m^2^) and weight gain as the difference of weight (kg) at the reference date and weight (kg) at age 18 years. Women with zero or negative weight gain were defined as having a zero weight gain.

Details of stage at diagnosis (*in situ*, localised, regional, distant) and lymph node involvement (number positive and number examined) were abstracted from the records at the CSP and at the hospital of diagnosis.

Follow-up began at interview and ended at the date of death, the date of last follow-up, or 30 June 2000, the end of the study period, whichever was earliest. Vital status and cause of death information was obtained routinely from CSP records, from computerised records of the California Death Certificate Master File and the National Death Index, and by contacting the patients or their relatives by telephone.

Of 969 eligible patients, we interviewed 744 (76.8%) but then excluded 11 postmenopausal patients, 11 with unknown cancer stage, and five deceased patients with unknown cause of death so that a total of 717 were available for analysis of BMI, weight and height. The first 199 patients enrolled in the case–control study did not receive the detailed assessment about lifetime exercise habits leaving 525 patients available for the exercise analyses. Of the 199 patients without detailed lifetime exercise data, 192 were determined to be otherwise eligible and were included in the analyses of body size and breast cancer survival; for these patients, we created a separate exercise activity code.

*χ*^2^ *P*-values were used to assess the relationship of patient characteristics at diagnosis with breast cancer survival. Hazard rate ratios (HR) and 95% confidence intervals (95% Cl) were calculated using Cox's proportional hazards methods (Breslow and Day, 1987). Events were deaths due to breast cancer; patients who died from other causes or were alive at the last follow-up were censored at the last follow-up date.

## RESULTS

A total of 454 interviewed patients (63.3%) were still alive at the date of last follow-up, with a median follow-up time of 10.4 years (Table 1

**Table 1:** Years of follow-up of premenopausal breast cancer patients aged 40 years and younger.

		**Years of follow-up**	
	**N**	**Median**	**Range**
All patients	717	10.4	0.3 –15.1
Alive at last follow-up	454	11.7	0.3 –15.1
Deceased due to breast cancer	251	4.5	0.6 –14.0
Deceased due to other causes^a^	12	9.0	1.0 –12.6

a Other causes of death included other cancers (*n*=7), coronary/cardiovascular disease (*n*=2), pulmonary disease (*n*=2), and infections (*n*=1).

). We identified 251 breast cancer deaths and 12 deaths due to other known causes among interviewed patients.

Nearly one-half of patients who died of breast cancer were age 35 years or younger at diagnosis compared to just over one-third of those who were alive at the last follow-up or died from other causes (Table 2

**Table 2:** Characteristics of eligible breast cancer patients aged 40 years or younger at diagnosis.

	**Still living or died from other known causes (*n*=466)**		**Died of breast cancer (*n*=251)**		
**Characteristics at breast cancer diagnosis**	***n***	**%**	N	**%**	**χ^2^ *P***
*Age (years)*					
21–25	6	1.3	11	4.4	
26–30	31	6.7	23	9.2	
31–35	132	28.3	85	33.9	
36–40	297	63.7	132	52.5	0.005
*Stage*					
*In Situ*	68	14.6	3	1.2	
Localised	255	54.7	85	33.9	
Regional	139	29.8	141	56.2	
Distant	4	0.9	22	8.7	<0.0001
*Axillary lymph node involvement*					
Yes	139	29.8	156	62.1	
No	323	69.3	88	35.1	<0.0001
Unknown	4	0.9	7	2.8	

). As expected, women who died of breast cancer were also more likely to have had an advanced stage and lymph node involvement at diagnosis than other patients. Almost two-thirds of patients who died of breast cancer had regional or distant disease at diagnosis compared to less than one-third of other patients; figures were similar for lymph node involvement.

We observed no clear association of lifetime exercise with risk of death from breast cancer, but higher levels of exercise in the year before the reference date were weakly associated with a of reduced risk of breast cancer death (Table 3

**Table 3:** Risk of dying from breast cancer in relation to physical activity from first menses to reference date and during year before the reference date, among breast cancer patients aged 40 years or younger at diagnosis

**Physical activity**		**Censored**	**Events**	**HR^b^**	**95% Cl**
*Average hours per week from first menses to reference date*					
0		117	68	1.00	
0.1–0.7		71	31	0.86	0.56–1.32
0.8–1.6		63	17	0.59	0.35–1.01
1.7–3.7		66	32	0.87	0.57–1.33
3.8+		35	25	1.30	0.81–2.09
Trend *P*				0.88	
					
*Average hours per week during year before reference date*					
0		265	143	1.00	
1–4		43	15	0.85	0.50–1.45
5+		44	15	0.78	0.45–1.34
Trend *P*				0.31	
Hrs/wk from first menses to reference date	Hrs/wk during year before reference date				
0	0	117	68	1.00	
0.1–3.7	0	135	63	0.86	0.61–1.21
3.8+	0	13	12	1.34	0.72–2.47
0.1–3.7	1+	65	17	0.61	0.36–1.04
3.8+	1+	22	13	1.26	0.68–2.32
Interaction *P*				0.07	

Hrs/wk=hours per week.

a Reference date is date one year before diagnosis.

b Models include age, stage at diagnosis and BMI.

). Compared to those who reported no exercise from their first menses to the reference date, women with the highest levels of activity did not experience a reduced risk of dying from breast cancer. However, compared to women who performed no exercise in the year before the reference date, those who performed at least 5 h of exercise per week, on average, experienced a 20% reduction in risk of breast cancer death; this was not statistically significant. We found no marked interaction between risk of death and various combinations of recent activity and lifetime activity levels.

We observed a nearly 25% reduced risk of death among women in the highest quartile of BMI compared to those in the lowest quartile of BMI, a finding that was not statistically significant (Table 4

**Table 4:** Body size indicators and risk of breast cancer death among breast cancer patients aged 40 years or younger at diagnosis

	**Censored**	**Events**	**HR^b^**	**95% Cl**
BMI (kg m^−2^)				
<20.4	110	65	1.00	
20.4–21.9	117	64	0.79	0.56–1.12
22.0–24.8	113	66	0.89	0.63–1.26
24.9+	118	64	0.76	0.53–1.07
Trend *P*			0.21	
				
Weight (kg)				
<54.1	116	57	1.00	
54.1–60.9	115	81	1.10	0.78–1.54
61.0–68.1	110	54	0.87	0.60–1.26
68.2+	125	59	0.86	0.60–1.23
Trend *P*			0.21	
				
Weight gain (kg)				
0	73	33	1.00	
0.1–5.0	181	112	0.97	0.66–1.43
5.1–10.0	104	49	0.81	0.52–1.24
10+	108	57	0.93	0.61–1.42
Trend *P*			0.53	
				
Height (m)				
<1.60	100	57	1.00	
1.60–1.64	133	68	0.92	0.65–1.30
1.65–1.68	124	59	0.84	0.58–1.21
1.69+	109	67	1.17	0.81–1.69
Trend *P*			0.52	

a Body size indicators at reference date (1 year before diagnosis), except for weight gain which was increase in weight from age 18 years to the reference age.

b Body size indicators at reference date (1 year before diagnosis), except for weight gain which was increase in weight from age 18 years to the reference age.

). Women in the highest quartile of weight experienced a statistically nonsignificant 14% reduction in risk of dying compared with women in the lowest quartile of weight. Weight gain from age 18 years to the reference date and height were not associated with risk of breast cancer death.

## DISCUSSION

Our findings are consistent with the only other study of physical activity and breast cancer survival which found no relation between risk of breast cancer death and total recreational activity performed in the year before diagnosis (Rohan *et al*, 1995), similar to our findings for lifetime exercise activity. Our median follow-up time (10.4 years) was nearly twice that of the earlier study (5.5 years), but even with the longer follow-up time we observed no marked association.

As we collected exercise activity data only for those who reported that they had either competed on a sports team, had serious and regular participation in dance, or had run or jogged regularly for at least one mile, some women who were recorded as having not participated in exercise activities may in fact have participated in some activities. The inclusion of these women in the reference category may bias the associations toward the null value, 1.0. However, the intent of the question was to identify the most active women, and activities among women who did not participate in these types of activities are likely to be limited.

Our finding of a weak association of BMI with decreased risk of death is somewhat inconsistent with the body of evidence. Approximately two-thirds of studies have reported an increasing risk of death with increasing BMI or weight, and the remaining studies generally reported no association (Goodwin and Boyd, 1990; Rock and Demark-Wahnefried, 2002). The women in the current study are among the youngest reported in the literature and as a group had a very low median BMI prior to diagnosis. It is possible that other factors, such as oestrogen receptor status, account for our findings, which are also consistent with no association.

A concern in this as in any follow-up study is loss to follow-up. The patients who were lost to follow-up early on may have differed from those with complete follow-up, leading to bias in the results. However, we were able to follow-up 79% of the patients to the end of the follow-up period and 92% to within 2 years of this point, so it is unlikely that such losses resulted in substantial biases. Another possible limitation is that recall of past exercise activities resulted in some misclassification, particularly those decades before interviews. However, we obtained activity information only from those who indicated that they had participated in serious sports activities or dancing, which allowed us to identify women engaged in strenuous exercise. Although some misclassification undoubtedly occurred, it is likely that our methods resulted in separation of the most active from the least active cohort members. Breast cancer diagnosis may have influenced recall of past exercise activities, but since all patients in the cohort were interviewed after learning their diagnoses, they should all have been similarly affected. All women in the cohort were interviewed prior to the widespread publication of papers indicating that exercise activity was associated with reduced breast cancer risk.

It is unclear whether prediagnosis physical activity levels are markers of postdiagnosis activity levels or whether postdiagnosis physical activity influences survival. A recent study reported 24–50% decreases in postdiagnosis compared to prediagnosis physical activity levels of breast cancer patients (Irwin *et al*, 2003), but postdiagnosis levels were assessed within 1 year of diagnosis. We interviewed 374 of the surviving breast cancer patients from this study in 1998–1999 as part of a follow-up study and found that, on average, surviving women had increased their activity levels in the years following their diagnoses.

Overall, we observe no association of lifetime exercise with breast cancer survival and only a weak association of higher levels of recent exercise with breast cancer survival. In contrast, we find a weak association of higher BMI levels with reduced risk of breast cancer death. It remains unclear whether recent, prediagnosis activity, and body size levels are indicators of postdiagnosis levels. Further examination of physical activity and body size during both pre- and postdiagnosis periods will improve our evaluation of the impact of these factors on breast cancer survival.
